# Study of the interactions of bovine serum albumin with a molybdenum(II) carbonyl complex by spectroscopic and molecular simulation methods

**DOI:** 10.1371/journal.pone.0204624

**Published:** 2018-09-27

**Authors:** Hélia F. Jeremias, Diana Lousa, Axel Hollmann, Ana C. Coelho, Carla S. Baltazar, João D. Seixas, Ana R. Marques, Nuno C. Santos, Carlos C. Romão, Cláudio M. Soares

**Affiliations:** 1 ITQB NOVA, Instituto de Tecnologia Química e Biológica António Xavier, Universidade Nova de Lisboa, Oeiras, Portugal; 2 Instituto de Medicina Molecular, Faculdade de Medicina, Universidade de Lisboa, Lisbon, Portugal; 3 Alfama Lda., Instituto de Biologia Experimental e Tecnológica, Oeiras, Portugal; University of Michigan, UNITED STATES

## Abstract

Therapy with inhaled carbon monoxide (CO) is being tested in human clinical trials, yet the alternative use of prodrugs, CO-Releasing Molecules (CORMs), is conceptually advantageous. These molecules are designed to release carbon monoxide in specific tissues, in response to some locally expressed stimulus, where CO can trigger a cytoprotective response. The design of such prodrugs, mostly metal carbonyl complexes, must consider their ADMET profiles, including their interaction with transport plasma proteins. However, the molecular details of this interaction remain elusive. To shed light into this matter, we focused on the CORM prototype [Mo(η^5^-Cp)(CH_2_COOH)(CO)_3_] (ALF414) and performed a detailed molecular characterization of its interaction with bovine serum albumin (BSA), using spectroscopic and computational methods. The experimental results show that ALF414 partially quenches the intrinsic fluorescence of BSA without changing its secondary structure. The interaction between BSA and ALF414 follows a dynamic quenching mechanism, indicating that no stable complex is formed between the protein Trp residues and ALF414. The molecular dynamics simulations are in good agreement with the experimental results and confirm the dynamic and unspecific character of the interaction between ALF414 and BSA. The simulations also provide important insights into the nature of the interactions of this CORM prototype with BSA, which are dominated by hydrophobic contacts, with a contribution from hydrogen bonding. This kind of information is useful for future CORM design.

## Introduction

The cytoprotective activity of induced heme oxygenase (HO-1) is a well-recognized property of this enzyme, which is induced to destroy toxic, free heme, liberated from injured or dead cells, thereby producing Fe^2+^ ions, bilirubin and free carbon monoxide (CO)[1]. The latter is a very important signaling molecule (often called gasotransmitter), akin to NO and H_2_S[2,3]. Importantly, applying CO from an external source elicits essentially the same physiological cytoprotective responses as HO-1, namely as an anti-inflammatory, anti-thrombotic and anti-proliferative agent.[4] This suggests that CO can be used as a therapeutic, as indeed demonstrated in animal models of vascular and inflammatory diseases, transplantation and a range of other indications[5,6]. However, the translation of this potential into real, practical CO-based therapy faces many hurdles[7].

The natural ability of hemoglobin (Hb) to scavenge CO strongly limits the use of CO inhalation as a useful form of treatment. Moreover, although used in animal models and human clinical trials, such method lacks tissue specificity and may require the use of high (toxic) CO concentrations in systemic circulation to produce biological effects[8]. Therefore, attempts to circumvent this difficulty have been tested using prodrugs known as CORMs (CO-Releasing Molecules). Most CORMs are transition-metal carbonyl complexes (MCCs), which decarbonylate *in vivo* more easily than other organic functions[9]. Ideally, CORMs must survive in circulation and target the organ in need, where they release CO in response to some locally expressed trigger[10–12]. Once it enters circulation, the MCC interacts with plasma proteins. Of these, the most abundant is serum albumin (SA) which functions as an important transporter of both endogenous and exogenous molecules in circulation,[13] thus determining several aspects of the pharmacokinetics of drugs[14,15].

The interaction of MCCs with SA may have three limiting scenarios: 1) the interaction involves extensive metal-ligand substitution accompanied by liberation of CO; 2) metal-ligand exchange is extensive but CO release is limited or much slower; 3) the interaction is non-covalent and CO is retained without bond breaking or bond formation. The first case is exemplified by [Mo(histidinate)(CO)_3_]Na (ALF186), which rapidly releases CO to the blood stream and does not show Mo-HEWL (HEWL = Hen Egg White Lysozyme) covalent interactions in model soaking experiments[16]. The second situation is best exemplified by the reaction of [Ru(CO)_3_Cl(glycinate)] (CORM-3) with both HEWL or BSA. In both cases one equivalent of CO is released as CO_2_, and adducts of [Ru(CO)_2_]^2+^ with the proteins are formed. The adduct BSA-Ru(CO)_2_[17,18], was confirmed to be responsible for the slow delivery of CO to targets *in vivo*[18]. Other examples are given by the formation of HEWL-M^I^(CO)_3_ (M^I^ = Mn^+^, Re^+^) from protein and [L_3_M(CO)_3_]^+^ complexes.[19,20] Such metal-ligand substitution reactions are similar to those observed with anti-cancer metal-based drugs devoid of CO ligands.[21] In the third situation, the non-covalent interaction of the MCC with the protein relies on intermolecular forces, including, in some cases, hydrogen bonds. In the absence of chemical reactions, such MCCs resist decomposition in the blood and do not increase COHb.

Experience tells us that M-CO bonds and organometallic ring ligands like π-cyclopentadienyl (π-Cp) impart marked lipophilic character to the MCCs. Therefore, they should interact with hydrophobic protein sites. However, such interactions are still not very well characterized. In fact, inert organometallic complexes are novel tools in Chemical Biology, where they have been successfully used as enzyme inhibitors and luminescent probes[22]. Although their interactions with specific enzyme binding sites have been studied, we are not aware of any study on the interactions of organometallic inert CORMs with plasma transport proteins. Information from such studies is important for the design of inert CORM prodrugs in terms of the prediction of their pharmacokinetic and ADMET parameters. Indeed, to be useful and to accomplish their therapeutic function, the interactions of the prodrug with SA must be well balanced. If they are too strong, the CORM may fail to exchange SA for the target disease tissue or may fail to react with the chemical or enzymatic triggers required for CO release [23,24]. If they are too weak, CORM’s half-life in circulation may decrease due to easy excretion or to detoxification happening before it is able to deliver its CO load.

**Fig 1:**
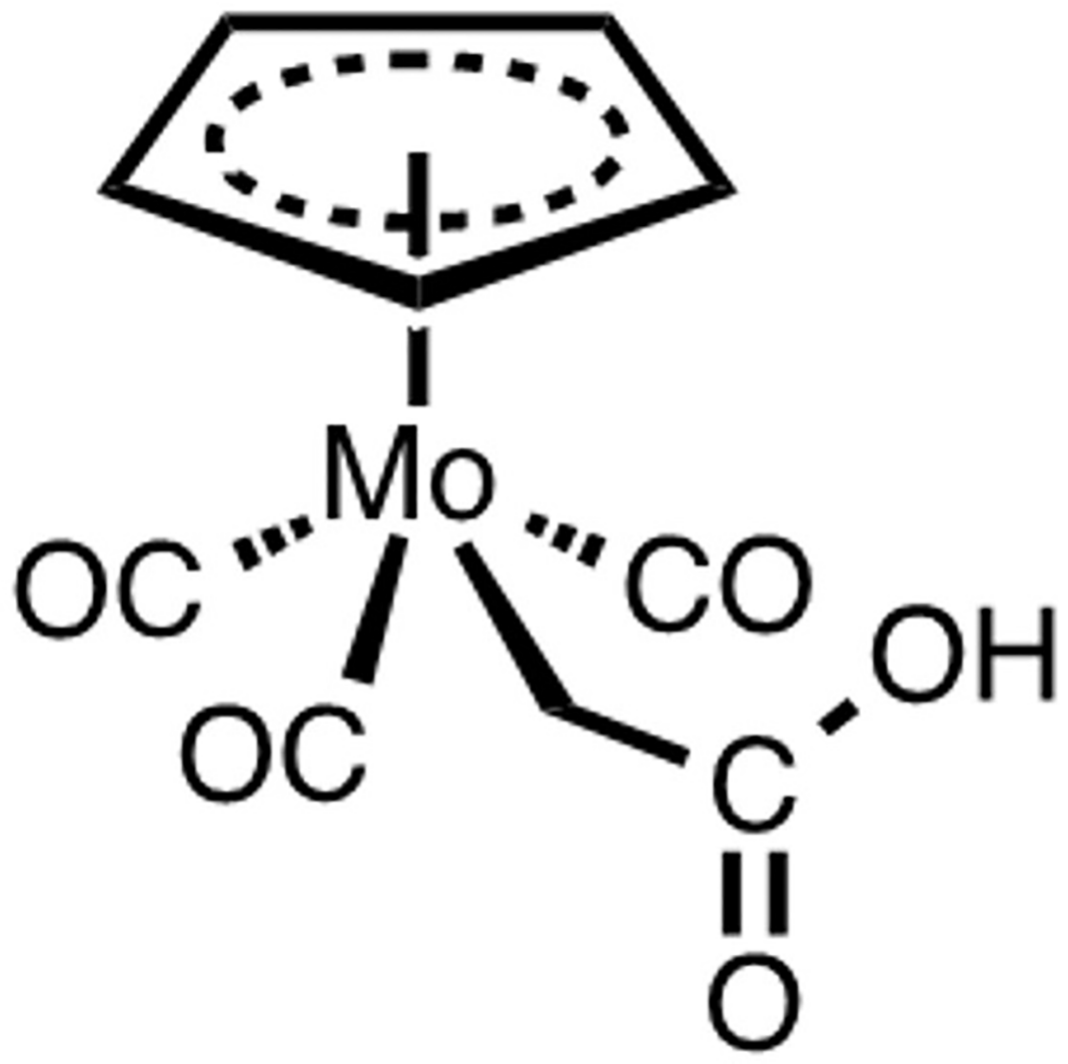
ALF414 structure.

In order to obtain fundamental information on the nature of such interactions, we selected [Mo(π-Cp)(CH_2_COOH)(CO)_3_] (ALF414; π-Cp = π-C_5_H_5_ = cyclopentadienyl)(Fig 1) as a model compound featuring a π-Cp ring, a substituted methyl ligand with a Mo-C σ-bond and three CO ligands. [25]. Adequately for our purposes, its globular 3D structure presents two hydrophobic regions, the aromatic π-Cp ring and the CO ligands, as well as a metallocarboxylic ligand that supports polar and H-bonding interactions. ALF414 does not undergo rapid thermally induced substitution reactions, except at high pH. It has never been studied *in vivo*, albeit several structurally related compounds have been reported as potentially useful CORMs.[26–30]

In the present work, we report spectroscopic (fluorescence spectroscopy and circular dichroism) and computational approaches to characterize the interactions of ALF414 with bovine serum albumin (BSA), which was chosen due to its low cost, availability and its 76% sequence homology with HSA. This type of fundamental information on the interaction of inert organometallic coordination spheres with serum albumin will certainly prove useful for the future design of pharmacologically improved CORMs.

## Results

ALF414 (Fig 1) belongs to the family of the so-called 4-legged piano stool complexes. Its low solubility in water is allegedly due to the formation of H-bridged dimers in the solid state, as shown in the X-ray crystal structure of the complex[25]. However, tests in concentrations ≤50μM could be performed in aqueous solution. Above this value, MeOH/H_2_O or DMSO/H_2_O mixtures were used as solvent. An unusual property of this carboxylic acid is its high p*K*_a_ value: 8.29±0.03 (determined in 1:1 H_2_O:dioxane). For comparison, the p*K*_a_ of acetic acid in water is 4.8. When dissolved at pH >10, ALF414 decomposes fairly quickly. It is stable in air for manipulation. It is also stable to serum, as no CO is released after 6 h when the compound is incubated with pure Fetal Bovine Serum (FBS), which has BSA as its main component.

ALF414 is not very cytotoxic, since, under the conditions of the MTT assay, the viability of HepG2 cells treated with ALF414 (100μM in 10% MeOH/H_2_O) for 24 h is 100%, whereas that of RAW 264.7 cells treated with ALF414 under the same conditions is 80%. Therefore, ALF414 has properties compatible with its potential use as an organometallic inert CORM. The fact that it was never used for this end *in vivo* is not relevant here since our focus is on the characterization of the interactions of serum albumin with a molecule with this kind of structure and ubiquitous ligands in its outer sphere. ALF414 is used here a prototype to study the interaction of this type of CORMs with serum albumin.

One important characteristic of ALF414 is the polarity of its ligand sphere. On one hand, it possesses the hydrophobic ligands CO and Cp, whereas on the other hand, it possesses a carboxylic function, which can support polar and H-bonding interactions with other biological molecules, namely proteins. In fact, from the intermolecular interaction point-of-view ALF414 is much closer to regular organic drugs than the labile, water soluble CORM-3 or ALF186 and its interactions with transport proteins should be amenable to study through the classical methods applied to small molecule organic drugs. This situation is important in the context of the optimization of organometallic CORMs, which have a globular type of structure[31,32] contrasting with the more common, relatively flat structures of most drugs.[31,32] To the best of our knowledge, no molecular interactions of this kind of CORMs with transport proteins have ever been reported. Likewise, molecular dynamic studies of MCCs with proteins are also absent from the literature.

### Fluorescence spectroscopy

**Fig 2:**
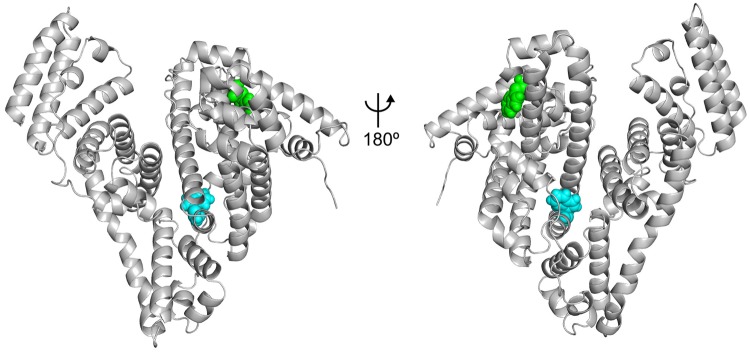
Location of the tryptophan residues on the structure of BSA. Residues Trp^134^ and Trp^213^ are represented as green and blue spheres, respectively.

Fluorescence quenching was used to evaluate the structural changes of BSA upon its interaction with ALF414. The intrinsic fluorescence of BSA is mainly due to its tryptophan (Trp) residues located in hydrophobic cavities in the subdomains IIA (Trp^213^) and IB (Trp^134^). The location of the Trp residues is shown in Fig 2.

**Fig 3:**
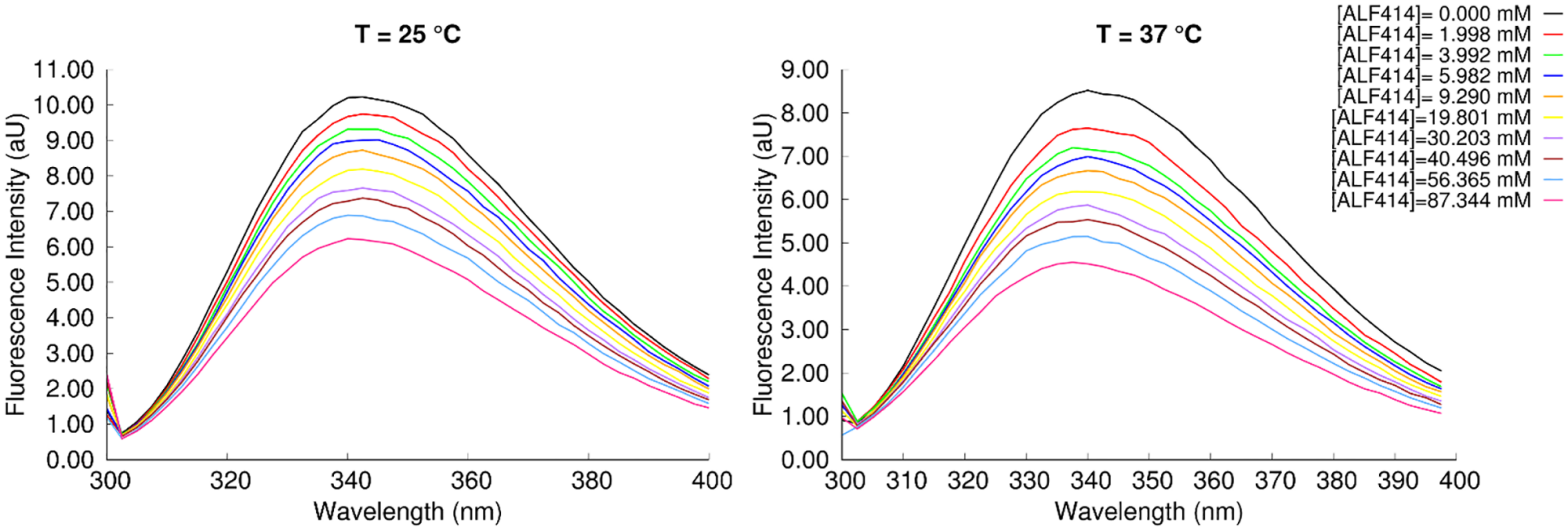
Intrinsic fluorescence emission spectra of BSA in the presence of ALF414 at 25°C and 37°C.

In general, the fluorescence quantum yield of Trp residues decreases with its increased exposure to the aqueous environment and proximity to polar molecules. In our experiments, BSA initial fluorescence intensity with emission at 340 nm (I_0_) was partially quenched by ALF414. Increased concentrations of ALF414 produced a reduction of fluorescence intensity, confirming the interaction of ALF414 with BSA (Fig 3).

**Fig 4:**
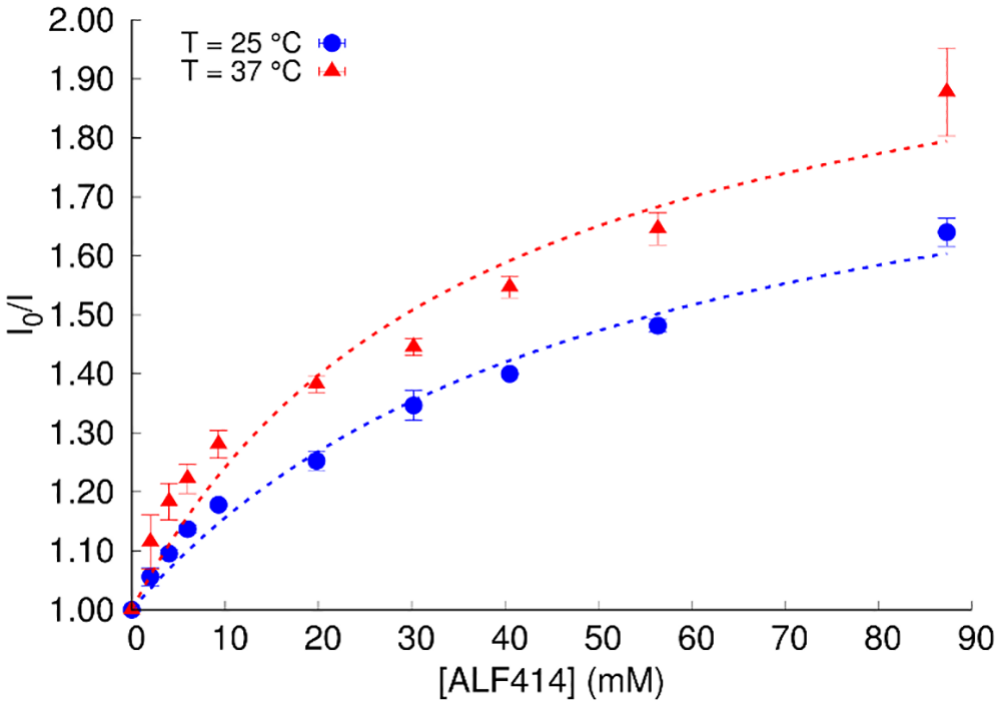
I_0_/I variation with increasing concentration of ALF414 at 25°C and 37°C.

Quenching mechanisms are usually classified as dynamic or static quenching. They can be distinguished by their different dependence on temperature and viscosity. Dynamic quenching depends upon diffusion. As higher temperatures result in larger diffusion coefficients, the bimolecular quenching constants are expected to increase with increasing temperature. In contrast, increased temperature is likely to result in decreased stability of complexes, and thus lower the values of the static quenching constants[33]. In this context, we evaluated the quenching by ALF14 at 25 and 37 °C. Importantly, the rise of I_0_/I is also steeper at 37 °C than at 25°C (Fig 4). The variation of I_0_/I with the concentration of ALF414 is not linear and deviates from the simple Stern-Volmer equation. Therefore data was analyzed using the Lehrer equation, which considers the existence of a subpopulation of the fluorophore accessible to the quencher and another inaccessible to it[34] (see S1 File for details).

In order to quantify the quenching efficiently, Stern-Volmer constants (K_sv_) were obtained by fitting the experimental data using that equation (Table A in S1 File). The significant difference (P ≤ 0.05) between K_sv_ value at 25°C (0.038 ± 0.003 μM^-1^) and 37°C (0.058 ± 0.001 μM^-1^) confirms a temperature-dependence on the quenching that suggests that the interaction between BSA and ALF414 follows a dynamic quenching rather than a static mechanism. This means that no stable complex is formed between the protein Trp residues (fluorophores) and ALF414 (quencher). Regardless of the temperature, *f*_*b*_ values (fraction of light emitted by the fluorophores accessible to the quencher (Table A in S1 File)) close to 0.5 were obtained (0.49 and 0.53 for 25°C and 37°C, respectively). This clearly shows that one of the two BSA Trp residues is accessible to the quencher, while the other is not. More importantly in the context of the objectives of the present work, these data indicate that upon binding to BSA, ALF414 interacts with one (and only one) of the protein’s Trp residues.

### Circular dichroism spectroscopy

**Fig 5:**
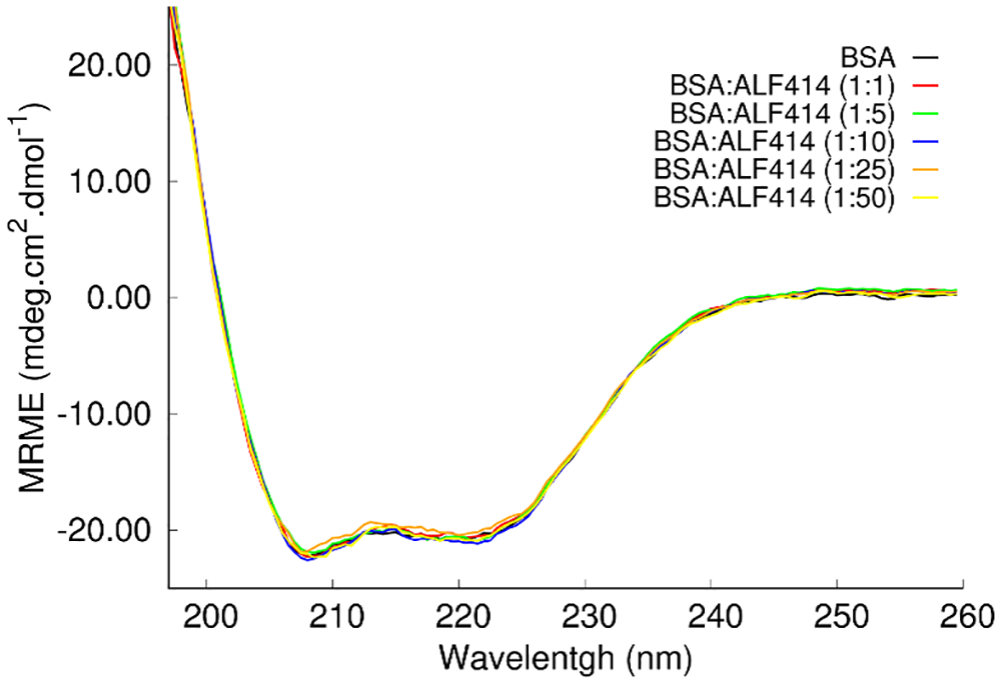
CD spectra of free BSA and BSA complexed with ALF414. The mean residue molar ellipticity (MRME) is plotted as a function of the wavelength.

Circular dichroism (CD) measurements were used to obtain information about the secondary structure of BSA and its changes upon binding of MCCs. Fig 5 shows the far-UV spectra of BSA in the absence and presence of several concentrations of ALF414. BSA in solution is mostly α-helical with two well-defined ellipticity values at 208 and 222 nm. These CD data show that there was no alteration in the BSA spectra with increasing concentrations of ALF414, indicating that the ligand interaction does not change the secondary structure of the protein.

### Simulation studies

Molecular docking was used to investigate the preferred binding mode of ALF414 to BSA and predict the affinity between these two molecules. In our specific case, we found many different binding sites scattered around the protein with similar energies (Fig A in S1 File), and, therefore, these results were not conclusive.

One of the largest limitations of the molecular docking method used here is the fact that the protein is considered rigid, which can compromise the accuracy of the predictions. In order to circumvent this limitation and take protein flexibility into account, we decided to use molecular dynamics (MD) simulations to understand the mechanism of interaction between the two molecules.

MD simulations of a system containing BSA and ALF414 in explicit aqueous solvent were performed, in which all the molecules were completely flexible. A large number of simulations (50) and ligands (25 in each simulation) were used to obtain statistically significant results. Visual inspection of the trajectories revealed that the ligand molecules rapidly approach the protein surface and cover most of it. This is not surprising, given that the ligands are quite hydrophobic at neutral pH. These molecules bind to many different regions, tumbling on the protein surface. It is not possible to detect an obvious preferred binding site by visual inspection of the trajectories. The comparison of the structural properties of BSA in the presence and absence of ALF414 showed that there are no significant changes on the protein nor that it changes upon interacting with the ligand (Fig C in S1 File).

**Fig 6:**
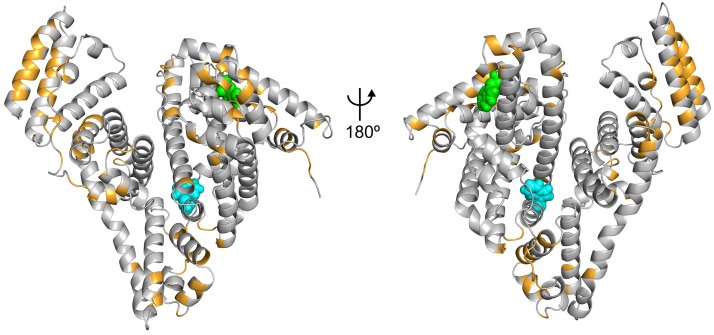
Binding sites of ALF414 on the surface of BSA. The residues that interact with the ligand for more than 30% of the total simulation time are highlighted in orange. Residues Trp^134^ and Trp^213^ are represented as green and blue spheres, respectively.

To find the preferred binding sites of ALF414, we analyzed the probability of interaction of each residue with this ligand (residue occupancy). As it can be observed in Fig 6, there are several residues of BSA that frequently interact with ALF414, which is consistent with the docking predictions. A sequence alignment of bovine and human serum albumin (Fig D in S1 File) shows that the regions of interaction with ALF414 found in the simulations are conserved between the two proteins (73% of the interacting residues are conserved and 10% are not conserved but have similar properties). This indicates that the results obtained are meaningful in a human context where the CORM molecules would be transported by HSA.

**Fig 7:**
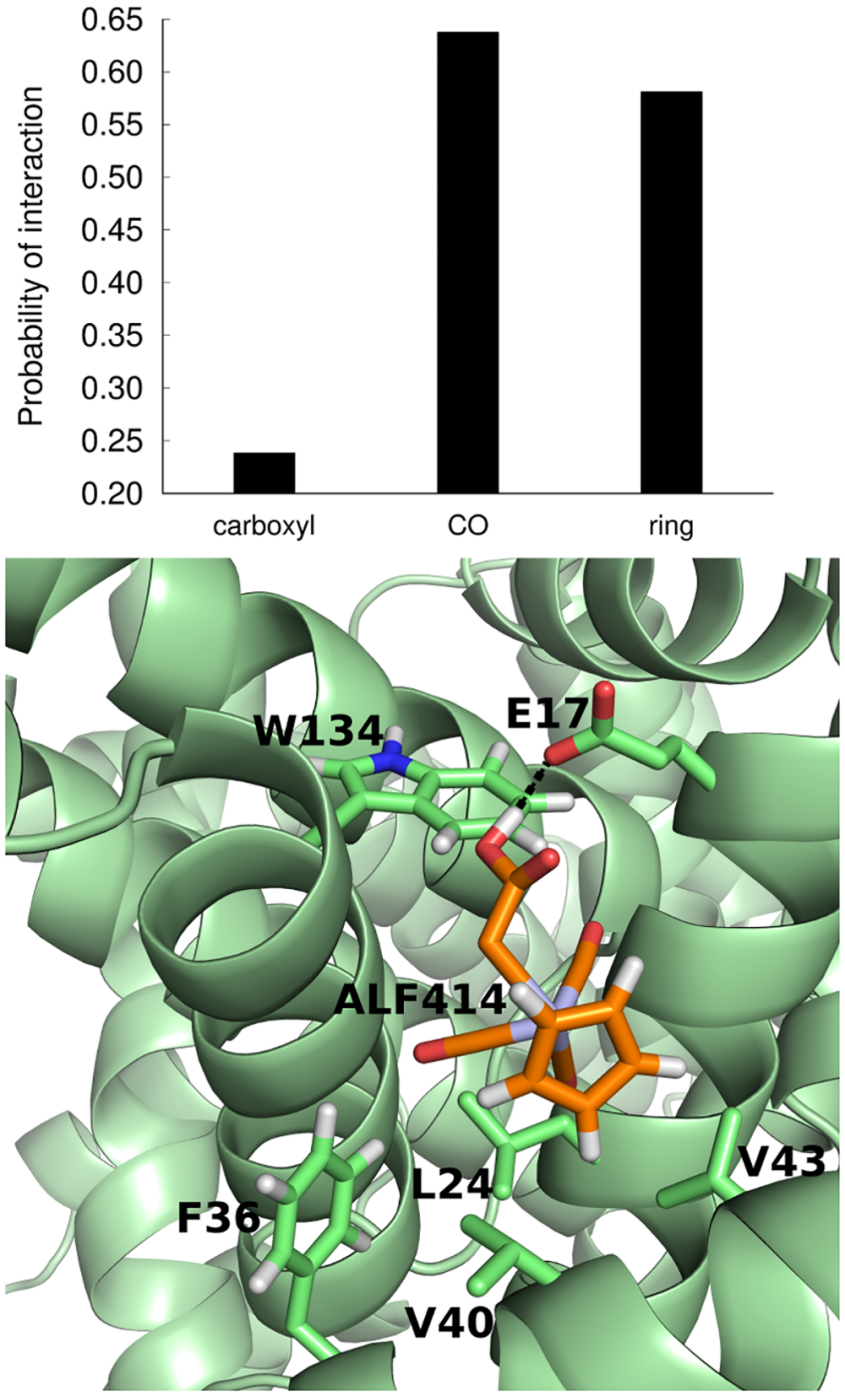
(A) Probability of interaction of the three moieties of ALF414 with BSA (B) Snapshot of replicate 7, where ALF414 is interacting with several residues, including Trp^134^, through hydrophobic contacts and hydrogen bonding.

One of the most populated binding sites contains a Trp residue (Trp^134^), which can explain the observed fluorescence quenching (a snapshot of a simulation where ALF414 is interacting with Trp^134^ is shown in Fig 7B). This result is also consistent with the docking calculations, which predicted that one of the preferred binding sites is located near Trp^134^. Both in the docking predictions and in MD simulations, only Trp^134^ and not Trp^213^ interacts with ALF414, which is in very good agreement with the fluorescence spectroscopy data indicating that only one Trp residue is quenched.

The MD simulation results also indicate that the interaction between Trp^134^ and ALF414 is quite dynamic and unstable (no stable complex is formed), as it can be observed in Movie_1. Once again, this observation is in line with the fluorescence analysis suggesting that the interaction between BSA and the ligand follows a dynamic quenching, rather than a static mechanism.

In order to determine the role played by each moiety of the ligand (Cp ring, carboxyl group and the CO groups), we analyzed their interaction with the protein separately (Fig 7A). The CO and ring moieties interact much more frequently with BSA than the carboxyl group. This shows that the interaction of this ligand with the protein is dominated by hydrophobic contacts, which is in line with previous studies focusing on the interaction of human serum albumin with anionic surfactants[35]. Hydrogen bonds between the carboxyl group and the protein also occur, although not very frequently (a snapshot of replicate 7, where ALF414 is interacting with several residues through hydrophobic contacts and hydrogen bonding, is shown in Fig 7B).

**Fig 8:**
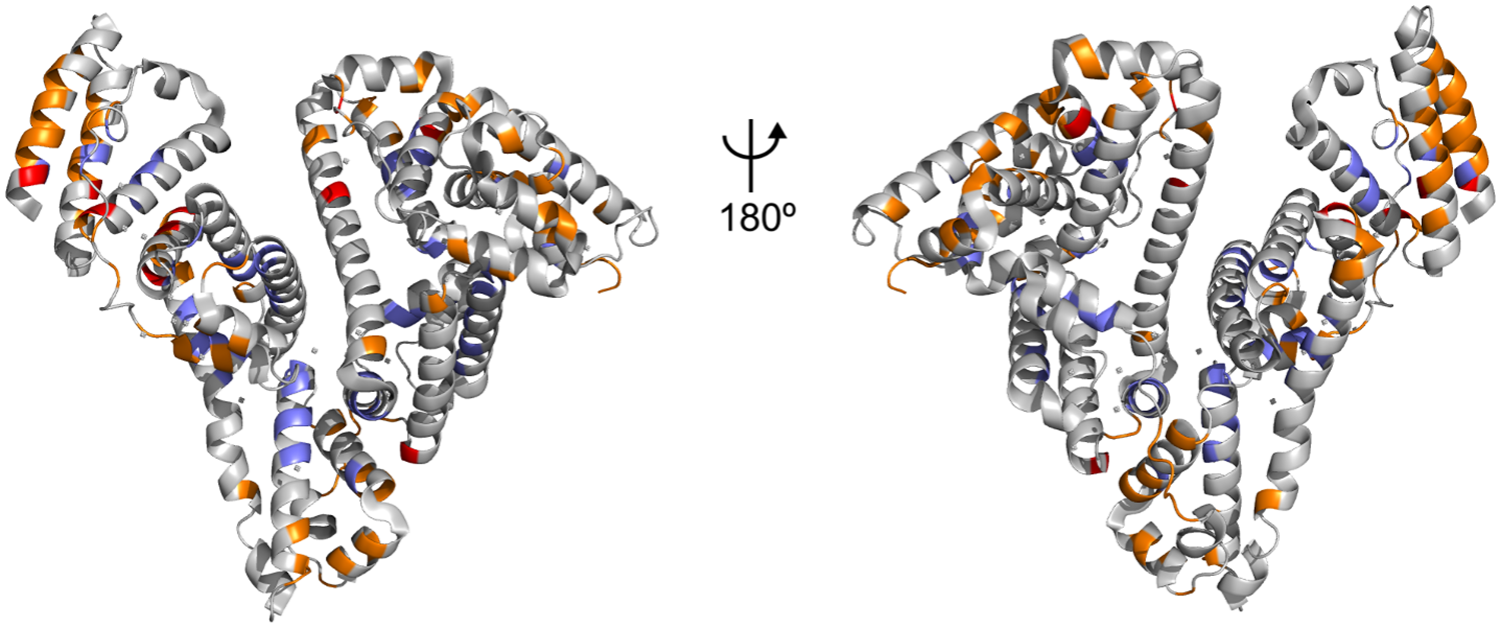
Comparison of the binding sites of ALF414 to BSA with the binding sites of hexadecanoic acid to HSA. The residues that interact with ALF414 in this work are colored in orange, the residues that bind hexadecanoic acid molecules are colored in purple and the residues that interact with both ligands are colored in red.

One of the biological roles of serum albumin is to transport molecules, including non-esterified fatty acids (FA). Crystallographic studies of HSA in the presence of FA have been used to identify the binding sites of these molecules and showed that there are seven FA-binding sites distributed throughout the protein[36]. The comparison of the binding sites of ALF414 to BSA with the binding sites of hexadecanoic acid to HSA (Fig 8) shows that some of the ALF414 binding sites are adjacent to the ones used by hexadecanoic acid, although only a few residues interact with both substrates. This is in line with the unspecific role of serum albumin, which can transport different molecules that tend to bind the hydrophobic pockets of the protein and in some cases also form polar interactions. This result is also consistent with previous studies showing that the protein uses the same binding sites to accommodate different ligands, although they do not always interact with the same residues. In fact, structural studies have shown that several drugs can co-bind the in the same region of the protein at the same time without overlapping, which in many cases creates a synergistic effect.[37]

Since ALF414 is bulkier and more globular than linear-chain fatty acids it does not have access to some of the most occluded binding sites used by these molecules.

## Discussion

The interaction of BSA with the CORM prototype ALF414, which belongs to the family of the so-called 4-legged piano stool complexes, was analyzed for the first time using a combination of spectroscopic and molecular simulation methods.

The analysis of the fluorescence emission spectra of BSA in the presence of increasing concentrations of ALF414 revealed that the initial fluorescence intensity of the protein was partially quenched by the ligand, which indicates that ALF414 interacts with at least one of the Trp residues of BSA, thus reducing the protein’s intrinsic fluorescence. This analysis further showed that the ligand interacts with only one of the two Trp residues of BSA, since the fraction of light emitted by the fluorophores accessible to the quencher was close to 0.5. Moreover, the temperature dependence of the quenching by ALF414 showed that it follows a dynamic mechanism, which indicates that the BSA-ALF414 interaction is dynamic. One could eventually reason that the fluorescence quenching, instead of being caused by a direct interaction with ALF414, could alternatively be due to a major conformational change of the protein induced by ALF414, leading to a complete change of the microenvironment of one of the Trp residues. However, this hypothesis is ruled out by the circular dichroism and molecular dynamics data, which indicates that there are no major changes in the BSA structure in the absence and presence of ALF414.

MD simulations indicated that ALF414 binds to different regions of the protein, including to one (and only one) of the Trp residues (Trp^134^), which is in agreement with the fluorescence results. The simulations revealed that ALF414 forms weak and dynamic interactions with BSA, i.e., it does not stay firmly bound to the binding sites. The comparison of the frequency of interaction of the different moieties of the ligand with the protein indicated that the CO and ring groups interact much more frequently with BSA than the carboxyl group.

The simulations showed that the structural properties of BSA in the absence and presence of ALF414 remain very similar (Fig C in S1 File), which means that the interaction with the ligand does not induce significant conformational changes on the protein. This is in agreement with the fact that the CD spectra of BSA in the presence of ALF414 remains unchanged relative to the spectra of the protein in solution. Since ALF414 binds weakly and transiently and does not react with the protein, it does not have a considerable effect on its structure. These interactions are totally different from those observed with the labile [Ru(CO)_3_] based CORMs, where there are reactions leading to covalent Ru-protein adducts.

Overall, this work provides a detailed and clear molecular description of the binding of ALF414 to BSA and represents the first study of this type on non-covalent CORM-protein interactions, combining experimental and computational methods. The experimental results and the molecular dynamics simulations are consistent and show that the interactions between ALF414 and BSA are dynamic and unspecific. They are mainly hydrophobic, with a small contribution from hydrogen bonding. Moreover, the simulation results also showed that ALF414 tends to use some of the same albumin binding sites that are used by other molecules, such as fatty acids.

ALF414 and several structurally related CORMs are rather thermally stable in the presence of proteins, yet they are sensitive to activation by reactive oxygen species—ROS—which are molecules containing oxygen that easily reacts with other molecules [38]. A molecule like ALF414 is expected to stay intact in circulation and breakdown only at the sites of inflammation where ROS species are overexpressed. Such expectation is supported by the fact that, as shown in the present work, ALF414 is neither sequestered nor activated by BSA, since it forms weak and transient interactions with the protein and there is no CO release in the presence of Fetal Bovine Serum. Sequestration could prevent it from reaching many inflamed tissues and cells whereas activation of ALF414 by plasma albumin would cause unproductive loss of CO to blood haemoglobin. We therefore expect liberation of CO from ALF414 to be “good enough” for anti-inflammatory activity, although this requires in vivo confirmation. CORMs with similar CO loads and activation processes have proven their beneficial CO delivery in liver inflammatory processes[23].

We believe that these results can be safely extended to other MCCs of the 3- and 4-legged piano stool types, several of which have been proposed as potentially useful CORMs[26,27,39]. These findings can be useful to understand the mechanism of interaction and transport of these small molecules binding to serum albumin and for the design of new drug-like CORMs that can be efficiently and safely delivered to the target cells.

## Materials and methods

ALF414 was prepared according to the literature and characterized by FTIR, ^1^H NMR, and elemental analysis (C,H,N)[25]. It can be handled in air but is best stored as solid under a nitrogen atmosphere in the dark. Microanalyses for CHN were performed at ITQB NOVA. NMR spectra were recorded on a Bruker Avance II 400 MHz spectrometer. FTIR spectra (KBr pellets) were taken in a Unicam-Mattson 7000.

### Stability studies

Stability in biological media was ascertained by monitoring the CO released from saturated mixtures of ≈ 3 mg/mL of ALF414 in pure Fetal Bovine Serum (FBS) in closed vials, in the dark, at 37°C. Samples of the headspace were collected using a Gastight Hamilton syringe and CO contents quantified by GC with a thermal conductivity detector as detailed elsewhere.[16]

### Cytotoxicity studies

Cells were incubated in a 96-well plate at 8×10^3^ cell/well, in a final volume of 180 μL. 24 h after cell seeding, 20 μL of the complex (in 10% MeOH/H_2_O) were added to each well, in order to have the respective final concentration in the well. 24 h after incubation with the respective complex, the medium was replaced with 100 μL of cell culture medium containing 1 mg/mL MTT. The cells were then incubated at 37 °C for 1 h. After removal of the cell medium, 100 μL of DMSO was added to dissolve the formed crystals. The plate was shaken for 5 min or until all the crystals were dissolved and the absorbance read at 550 nm using a BioRad microplate reader.

The cytotoxicity of ALF414 was evaluated on RAW264.7 (mouse monocyte macrophages—ECACC 91062702) and HepG2 cells (human hepatoma cell line—ECACC85011430).

### Fluorescence spectroscopy

Fluorescence spectra were recorded on a Varian Cary Eclipse fluorescence spectrophotometer, using 1.0 cm quartz cells. Excitation and emission slits were fixed at 2.5 and 5 nm, respectively. The excitation wavelength was set to 295 nm to selectively excite tryptophan residues, and the emission spectra were recorded at 25°C and 37°C, in the of 300–400 nm range at a scan rate of 300 nm.s^-1^. The fluorescence intensity of the complexes in buffer was negligible under these conditions. BSA concentration was kept at 2.0 μM in all experiments, and aliquots of each solution of compounds from a 2.0 mM stock solution were added successively. Fluorescence intensity was measured 10 min after each addition, at the λ_max_. After inner filter correction, data analysis was done using the Stern-Volmer and Lehrer equations[34,40] (See S1 File).

### Circular dichroism (CD) spectroscopy

CD spectra of free BSA and the complex between MCCs and BSA were recorded in the 195–260 nm range on a Jasco J-815 spectrometer, at 25°C, using 1 mm quartz cells. Each sample was scanned three times at 100 nm.s^-1^, using a 1 s time constant, and a bandwidth of 1.0 nm. The concentration of BSA was kept at 3 μM in the solution. The PBS buffer solution was used as blank and the baseline was corrected. The CD spectra of all the compounds were recorded and no CD signal was obtained with ALF414 alone. The CD signal was converted to mean residual ellipticity, θ, in mdeg.cm^2^.dmol^-1^, according to ref. [41]

### Molecular docking of ALF414 to BSA

Molecular docking simulations of ALF414 were performed using the AutoDock 4.5 program with the Lamarckian genetic algorithm[42]. The details of the docking calculations can be found in S1 File.

### Molecular dynamics (MD) simulations

The simulations were performed using GROMACS 4.5.5[43] and the united atom GROMOS54A7 force field[44] for the protein and the SPC model[45] for water. The parameters for AlF414 were derived using the protocol described in the S1 File. The temperature and pressure were kept constant at 300 K and 1 atm, respectively, using the Berendsen algorithm[46], with a temperature coupling constant of 0.1 ps^-1^, with separate heat baths for the protein and the rest of the system, and a pressure coupling constant of 0.5 ps^-1^.

A cut-off of 0.9 nm was used for electrostatic interactions and the Particle Mesh Ewald algorithm[47] was used to treat electrostatic interactions beyond this cut-off. Van der Walls interactions were calculated up to 1.4 nm. The solute bonds were constrained with the LINCS algorithm[48] and SETTLE[49] was used for the solvent. Fifty MD simulations of 30 ns were computed, each containing one copy of BSA and twenty-five copies of the ligand in solution. To prevent the ligand molecules from sticking to each other, the Lennard-Jones parameter C12 for Mo-Mo interactions was increased to 7.98×10^−1^.

The simulations were started from the X-ray structure of BSA (PDB code 4F5S) and the protonation states predicted using a methodology based on Poisson Boltzmann/Monte Carlo calculations[50,51] and considering a pH of 7.

The protein was solvated in a dodecahedral box with 31,560 water molecules, twenty-five copies of ALF414 and six ions, defining a minimum distance of 1.0 nm between the protein and the box walls. We performed three energy minimization steps using the steepest descent algorithm. In the first step, all atoms were restrained with a force constant of 1 KJ/mol Å^2^ except the hydrogen atoms. In the second step, only C-α atoms were restrained with the same force constant and in the last step no restrains were used. The simulations were initialized using a three-step procedure. In the first 50 ps of simulation, velocities were generated from a Boltzmann distribution at 300 K, all the heavy atoms were restrained and the simulation was performed in the NVT ensemble with a temperature coupling constant of 0.05 ps^-1^. This was followed by 50 ps of simulation in the NPT ensemble, with temperature and pressure coupling constants of 0.05 and 0.5 ps^-1^, respectively, maintaining the restraints on the heavy atoms. In the following 100 ps of simulation, only the C-alpha carbons were restrained and the temperature coupling was changed to 0.1 ps^-1^. After this procedure, all the atoms were released and the simulations were extended for 30 ns.

## Supporting information

S1 File Supporting information material. This file contains all supporting information data, methods, figures and tables.
(DOCX)
