# Supplementary material for: Study of the interactions of bovine serum albumin with a molybdenum(II) carbonyl complex by spectroscopic and molecular simulation methods
Source: PLoS One. 2018 Sep 27;13(9):e0204624. doi: 10.1371/journal.pone.0204624 (PMC6160121; doi:10.1371/journal.pone.0204624)
Supplement: S1 File — This file contains all supporting information data, methods, figures and tables. (DOCX) [file pone.0204624.s001.docx]

Supporting Information for the manuscript

**Study of the interactions of bovine serum albumin with a molybdenum(II) carbonyl complex by spectroscopic and molecular simulation methods**

Hélia F. Jeremias^a^‡, Diana Lousa^a^‡, Axel Hollmann^b^, Ana C. Coelho^a^, Carla S. Baltazar^a^, João D. Seixas^c^, Ana R. Marques^c^, Nuno C. Santos^b^, Carlos C. Romão^a, c^* and Cláudio M. Soares^a^*

^a^ ITQB NOVA, Instituto de Tecnologia Química e Biológica António Xavier, Av. da República, 2780-157, Oeiras, Portugal

^b^Instituto de Medicina Molecular, Faculdade de Medicina, Universidade de Lisboa, Av. Prof. Egas Moniz, 1649-028, Lisbon, Portugal

^c^Alfama Lda., Instituto de Biologia Experimental e Tecnológica, Av da República, 2780-157, Oeiras, Portugal

**Fluorescence Studies**

The Stern-Volmer equation is presented below (eq. 1). *I*_0_ is the fluorescence intensity of the fluorophore in the absence of quencher, *I* is the fluorescence intensity at variable quencher concentrations [Q] and K_SV_ is the Stern-Volmer constant.

$\frac{I_{0}}{I}=1+K_{sv}\left[ Q \right]$ (eq 1)

The Lehrer equation, an altered form of the classical Stern-Volmer relation, is used when the protein studied contains more than one fluorophore population, where *f*_B_ is the fraction of light emitted by the fluorophores accessible to the quencher (equation 2)

$\frac{I_{0}}{I}=\frac{1+K_{sv}\left[ Q \right]}{\left( 1+K_{sv}\left[ Q \right] \right)\left( 1-f_{B} \right)+f_{B}}$ (eq. 2)

Table A. Quenching constants of BSA by ALF414, obtained by fitting the experimental data (Fig 2) with equation (2).

|  | **T (°C)** | **K_sv_ (µM^-1^)** | $\boldsymbol{f}_{\boldsymbol{B}}$ |
| --- | --- | --- | --- |
| **ALF414** | 25  37 | 0.038  0.058 | 0.49  0.53 |

Molecular docking of ALF414 to BSA

Molecular docking simulations of ALF414 were performed using the AutoDock 4.5 program, with the Lamarckian genetic algorithm^1^. The BSA crystal structure was retrieved from the protein data bank (PDB ID: 4F5S)^2^. All waters were removed from the structure and only polar hydrogens were considered. Docking used a grid box of 82.5 × 82.8 × 86.25 Å^3^ with 0.375 Å spacing, covering all the protein. All ALF414 torsional bonds were considered as free. In addition, BSA was considered as rigid. Ten thousand docking runs for ALF414 were conducted with a population of 150 random individuals, and a maximum number of 1×10^6^ energy evaluations. All other parameters were performed as described in ref. ^3^. A root mean square of 2.0 Å was used for clustering.

The results obtained in the docking calculations are shown in figure S4. The figure shows that the docking method finds many different binding sites with similar energy values. In one of the preferred binding modes the ligand is interacting with Trp^134^.


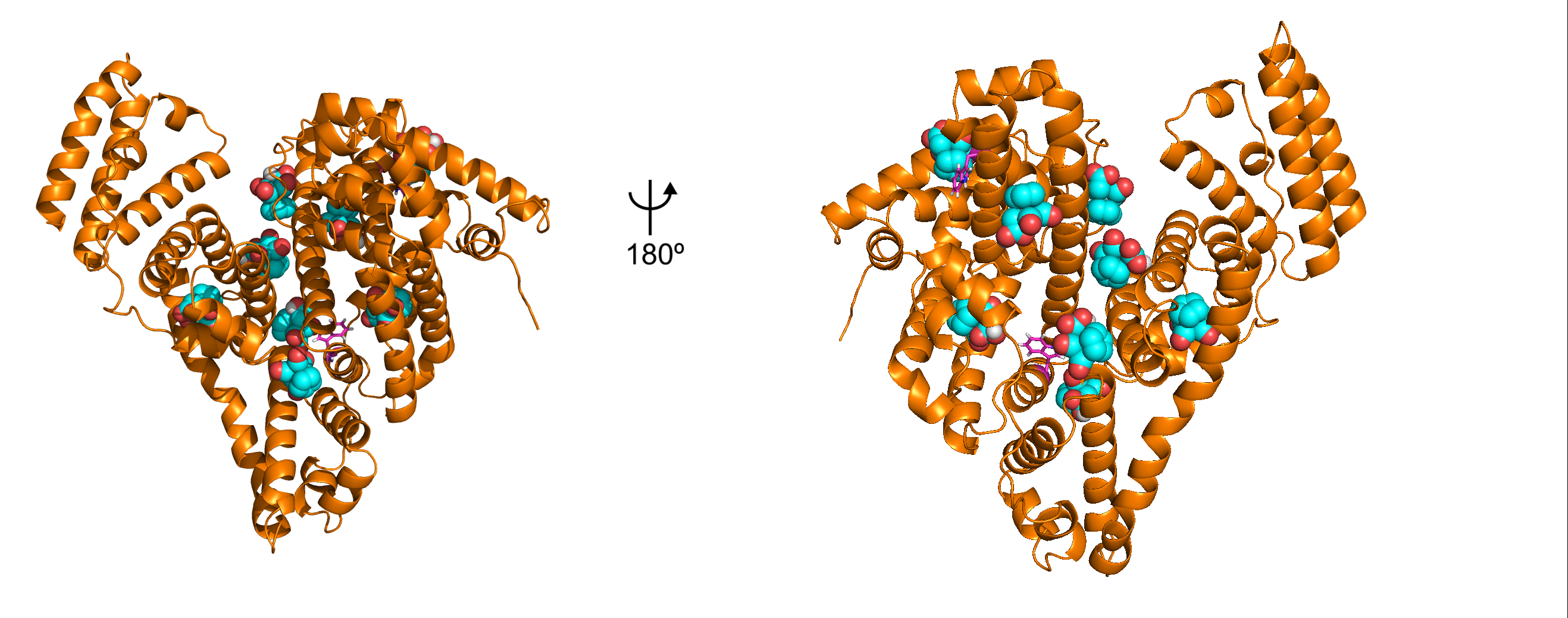


Fig A. Binding modes of ALF414 to BSA obtained in the docking calculations. The ligand conformations corresponding to docking solutions with binding free energies between -6 kcal/mol and -5 kcal/mol are shown using spheres with carbons colored in cyan. The two Trp residues of BSA are represented using magenta sticks.

Parameterization of ALF414

The carboxylic acid moiety of ALF414 was considered protonated, since its pKa is 8.29±0.03 and we are trying to reproduce biological conditions (pH ~7). The atomic partial charges for ALF414 (Figure S3) were calculated by Restrained ElectroStatic Potential (RESP) fitting^4^ on electrostatic potentials calculated with GAUSSIAN09^5^. The molecule was energy optimized using B3LYP, the cc-pVTZ basis set for organic atoms and the SDD effective core potential (ECP) for molybdenum (Mo). PCM was used to account for solvent effects, considering a dielectric constant of 4. The final geometry corresponded to a minimum, as verified by a frequency calculation, and was used to generate electrostatic potentials in space.


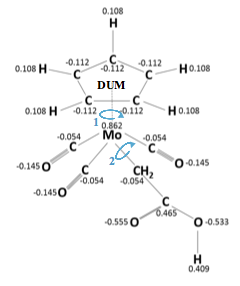


Fig B. ALF414 partial atomic charges.

Lennard-Jones parameters for Mo, to be used in MD simulations, were adapted from the Universal Force Field^6^. The parameters for the bond and angle interactions were adapted from the GROMOS 54A7 force field^7^. We created a dummy atom in the geometric center of the Cp ring in order to connect the ring to the Mo(CO)_3_CH_2_COOH moiety, instead of connecting Mo directly to the carbon atoms of the Cp ring. This allows for the rotation of the Cp ring with respect to the Mo(CO)_3_CH_2_COOH moiety, and is used for reasons of parameterization within the realm of the empirical molecular mechanics force field (where we account for the Mo-Cp interactions using a dihedral). To parameterize the C(ring) – DUM – Mo – CH_2_ and DUM – Mo – CH_2_ – COOH dihedrals (represented as blue arrows in Figure S3), we fitted the empirical potential energy to the quantum chemical potential obtained with Gaussian. Single point calculations on the different molecular conformations (generated by geometric rotation of the different dihedrals) were done using MP2 and the cc-pVTZ basis set for organic atoms and the SDD effective core potential (ECP) for Mo. PCM was also used to account for solvent effects, considering a dielectric constant of 4. For both torsions 1 and 2 (shown as blue arrows in Figure S3), the quantum potential was fitted to a linear combination of terms with the GROMACS functional form, with the parameters given in Table S2. In practice, this means that each of these torsions is modelled by a sum of dihedral terms.

Table B. Dihedral parameters

| Torsion | Dihedral term | Phase shift | Multiplicity | Force constant |
| --- | --- | --- | --- | --- |
| 1 | 1 | 105.181 | 5 | 2.47 |
|  | 2 | -135.014 | 4 | 0.18 |
|  | 3 | -138.106 | 10 | 0.26 |
| 2 | 1 | -102.841 | 1 | 12.63 |
|  | 2 | 46.5048 | 2 | 5.21 |
|  | 3 | 161.461 | 3 | 8.44 |
|  | 4 | -117.57 | 4 | 1.28 |
|  | 5 | 55.2939 | 5 | 1.97 |
|  | 6 | -261.971 | 6 | 2.01 |
|  | 7 | -287.351 | 8 | 0.73 |

**Effect of ALF414 on the structural properties of BSA**

Comparison of the structural properties of BSA in the presence and absence of ALF414 showed that the protein does not change upon interaction with the ligand (fig. S4). We note that the high RMSD values displayed by BSA are not due to protein unfolding, but rather correspond to inter-domain movements of the protein. Five replicate simulations of free BSA were performed, whereas for the simulations performed with BSA and ALF414 we used 50 replicates, since a good sampling was required to analyse the protein-ligand interactions. In order to make an unbiased comparison of the two conditions, we are showing only the first five replicates of BSA with ALF414 in the plots below.

Free BSA

BSA with ALF414

**
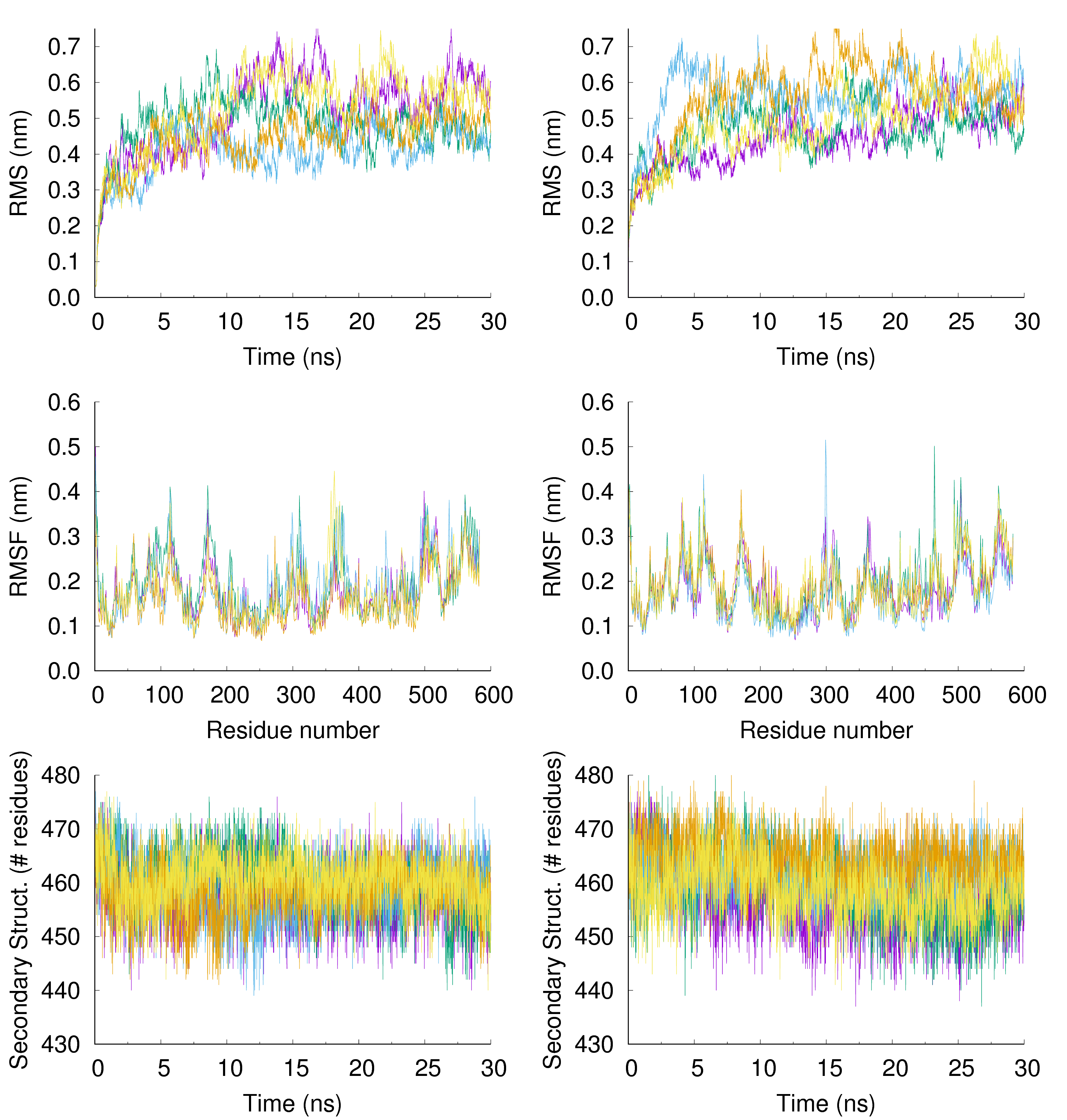
**

**Fig C.** Structural properties of BSA in MD simulations performed in the absence (left side) and presence (right side) of ALF414. Each replicate simulation is displayed in a different colour. The plots in the third row correspond to the total secondary structure content of the protein**.**

**Comparison of bovine and human serum albumin**

BSA            1 DTHKSEIAHRFKDLGEEHFKGLVLIAFSQYLQQCPFDEHVKLVNELTEFA 50

HSA            1 DAHKSEVAHRFKDLGEENFKALVLIAFAQYLQQCPFEDHVKLVNEVTEFA 50

BSA           51 KTCVADESHAGCEKSLHTLFGDELCKVASLRETYGDMADCCEKQEPERNE 100

HSA           51 KTCVADESAENCDKSLHTLFGDKLCTVATLRETYGEMADCCAKQEPERNE 100

BSA           101 CFLSHKDDSPDLPKL-KPDPNTLCDEFKADEKKFWGKYLYEIARRHPYFY 149

HSA           101 CFLQHKDDNPNLPRLVRPEVDVMCTAFHDNEETFLKKYLYEIARRHPYFY 150

BSA           150 APELLYYANKYNGVFQECCQAEDKGACLLPKIETMREKVLTSSARQRLRC 199

HSA           151 APELLFFAKRYKAAFTECCQAADKAACLLPKLDELRDEGKASSAKQRLKC 200

BSA           200 ASIQKFGERALKAWSVARLSQKFPKAEFVEVTKLVTDLTKVHKECCHGDL 249

HSA           201 ASLQKFGERAFKAWAVARLSQRFPKAEFAEVSKLVTDLTKVHTECCHGDL 250

BSA           250 LECADDRADLAKYICDNQDTISSKLKECCDKPLLEKSHCIAEVEKDAIPE 299

HSA           251 LECADDRADLAKYICENQDSISSKLKECCEKPLLEKSHCIAEVENDEMPA 300

BSA           300 NLPPLTADFAEDKDVCKNYQEAKDAFLGSFLYEYSRRHPEYAVSVLLRLA 349

HSA           301 DLPSLAADFVESKDVCKNYAEAKDVFLGMFLYEYARRHPDYSVVLLLRLA 350

BSA           350 KEYEATLEECCAKDDPHACYSTVFDKLKHLVDEPQNLIKQNCDQFEKLGE 399

HSA           351 KTYETTLEKCCAAADPHECYAKVFDEFKPLVEEPQNLIKQNCELFEQLGE 400

BSA           400 YGFQNALIVRYTRKVPQVSTPTLVEVSRSLGKVGTRCCTKPESERMPCTE 449

HSA           401 YKFQNALLVRYTKKVPQVSTPTLVEVSRNLGKVGSKCCKHPEAKRMPCAE 450

BSA           450 DYLSLILNRLCVLHEKTPVSEKVTKCCTESLVNRRPCFSALTPDETYVPK 499

HSA           451 DYLSVVLNQLCVLHEKTPVSDRVTKCCTESLVNRRPCFSALEVDETYVPK 500

BSA           500 AFDEKLFTFHADICTLPDTEKQIKKQTALVELLKHKPKATEEQLKTVMEN 549

HSA           501 EFNAETFTFHADICTLSEKERQIKKQTALVELVKHKPKATKEQLKAVMDD 550

BSA           550 FVAFVDKCCAADDKEACFAVEGPKLVVSTQTALA- 583

HSA           551 FAAFVEKCCKADDKETCFAEEGKLKVAASQAALGL 585

**Fig D.** Sequence alignment of bovine serum albumin (BSA) with human serum albumin (HSA) generated by EMBOSS Needle using the EBLOSUM62 matrix with a gap penalty of 10.0 and an extend penalty of 0.5. The residues that interact with ALF414 in MD simulations are highlighted using green for residues which are conserved between the two proteins, blue for residues which are not conserved but have similar physical chemical properties, and yellow for residues that have different physical chemical properties.

**References**

1. Morris, G. M.; Huey, R.; Lindstrom, W.; Sanner, M. F.; Belew, R. K.; Goodsell, D. S.; Olson, A. J. Autodock4 and Autodocktools4: Automated Docking with Selective Receptor Flexibility. *J. Comp. Chem.* **2009**, *30*, 2785-2791.

2. Bujacz, A. Structures of Bovine, Equine and Leporine Serum Albumin. *Acta Crystallographica Section D* **2012**, *68*, 1278-1289.

3. Lousa, D.; Baptista, A. M.; Soares, C. M. Structural Determinants of Ligand Imprinting: A Molecular Dynamics Simulation Study of Subtilisin in Aqueous and Apolar Solvents. *Prot. Sci.* **2011**, *20*, 379-386.

4. Bayly, C. I.; Cieplak, P.; Cornell, W. D.; Kollman, P. A. A Well-Behaved Electrostatic Potential Based Method Using Charge Restraints for Deriving Atomic Charges - the Resp Model. *J. Phys. Chem.* **1993**, *97*, 10269-10280.

5. Frisch, M. J.; Trucks, G. W.; Schlegel, H. B.; Scuseria, G. E., *Gaussian 09. 2009, Gaussian*. 2009: Inc.: Wallingford CT.

6. Rappe, A. K.; Casewit, C. J.; Colwell, K. S.; Goddard, W. A.; Skiff, W. M. Uff, a Full Periodic-Table Force-Field for Molecular Mechanics and Molecular-Dynamics Simulations. *J. Am. Chem. Soc.* **1992**, *114*, 10024-10035.

7. Schmid, N.; Eichenberger, A. P.; Choutko, A.; Riniker, S.; Winger, M.; Mark, A. E.; van Gunsteren, W. F. Definition and Testing of the Gromos Force-Field Versions 54a7 and 54b7. *Eur. Biophys. J.* **2011**, *40*, 843-856.
